# Association Between Pneumoconiosis and Pleural Empyema: A Retrospective Cohort Study

**DOI:** 10.3390/diagnostics15233075

**Published:** 2025-12-03

**Authors:** Khay-Seng Soh, Cheng-Li Lin, Wei-Ming Lee, Der-Yang Cho

**Affiliations:** 1 Department of Veterinary Medicine, National Chung Hsing University, No. 145, Xingda Road, Taichung 402, Taiwan; 2 Department of Surgery, China Medical University Hospital, Taichung 404, Taiwan; 3 Department of Surgery, Chu Shang Show Chwan Hospital, Nantou 557, Taiwan; 4 Management Office for Health Data, China Medical University Hospital, Taichung 404, Taiwan

**Keywords:** pneumoconiosis, occupational disease, pleural empyema

## Abstract

**Background:** Pneumoconiosis is associated with chronic pulmonary and systemic complications, yet its relationship with pleural empyema remains insufficiently defined. This study evaluated the risk of pleural empyema among individuals with pneumoconiosis using a nationwide population-based cohort. **Methods:** Using Taiwan’s National Health Insurance database, we identified 14,441 patients with newly diagnosed pneumoconiosis and 57,764 matched controls by age, sex, and index year. Pleural empyema was ascertained using ICD-based definitions. Cox proportional hazards models, stratified and cluster-adjusted to account for matched design, were used to estimate hazard ratios (HRs). Competing-risk models, propensity score matching, E-value estimation, and mediation analysis were performed to evaluate robustness and residual confounding. **Results:** During follow-up, the incidence of pleural empyema was significantly higher in the pneumoconiosis cohort than in controls (2.33 vs. 1.02 per 1000 person-years). Pneumoconiosis was associated with an increased risk of pleural empyema (adjusted HR = 1.79, 95% CI: 1.47–2.18), consistent across subgroup analyses and competing-risk models. The strongest associations were observed among younger individuals and those without comorbidities. Sensitivity analyses, including 1:1 propensity score matching, yielded similar effect estimates. The E-value suggested that only a strong unmeasured confounder could fully explain the observed association. **Conclusions:** Patients with pneumoconiosis face a substantially elevated risk of developing pleural empyema, independent of demographic factors, comorbidities, corticosteroid use, and healthcare utilization. Intensified monitoring for pleural complications in pneumoconiosis patients who develop respiratory infections may lead to earlier diagnosis and a reduction in the negative outcomes associated with pleural empyema.

## 1. Introduction

Pneumoconiosis represents a heterogeneous group of chronic lung diseases triggered by the long-term inhalation of dust particles, including coal dust, silica, asbestos, and various other substances. Characterized by persistent pulmonary inflammation and progressive fibrosis, it results in impaired respiratory function and an increased risk of death [1]. While industrial regulations and awareness of workplace safety have improved, pneumoconiosis persists as a major public health issue, particularly affecting aging individuals who were historically exposed to hazardous occupational settings.

A comprehensive Taiwanese study reported that within a cohort of 37,281 workers with occupational exposure to dust, 1812 individuals received a diagnosis of pneumoconiosis (4.9%). The highest prevalence was observed among coal miners (6.2%), followed by refractory brick workers (5.5%) and manufacturers of asbestos products (4.4%) [2]. Despite a downward trend in the incidence rate in recent years, attributable to the contraction of the mining sector and enhanced workplace safety protocols, a substantial proportion of affected individuals have now advanced in age, leading to an escalating demand for long-term medical care.

On a global scale, pneumoconiosis continues to contribute to the overall disease burden, with an estimated prevalence of 520,000 cases and an annual incidence of 60,000 new cases, culminating in approximately 20,000 deaths annually [3,4]. Pneumoconiosis is recognized to be associated with a range of comorbid conditions, encompassing pulmonary fibrosis [5], chronic obstructive pulmonary disease (COPD) [5], pneumothorax [6], congestive heart failure [7], atrial fibrillation [8], coronary artery disease [9], peripheral artery disease [10], and cerebrovascular disease [11,12]. These associations underscore the systemic ramifications and the clinical imperative for early detection and management strategies [13,14,15].

Available evidence supports a plausible association between pneumoconiosis and pleural empyema—a grave complication characterized by the collection of purulent exudates within the pleural cavity, most often as a consequence of pneumonia [16,17]. Pneumoconiosis-related structural alterations in the pleura, such as pleural thickening and effusion, may impede fluid resorption mechanisms and augment susceptibility to infection [18,19,20]. Investigations have revealed that up to 58% of patients with pneumoconiosis exhibit pleural thickening, and 11% present with pleural effusion, indicating that pleural involvement is not uncommon [19]. Furthermore, compromised macrophage function, impaired pulmonary immunity, and chronic inflammation may render these patients more vulnerable to recurrent respiratory infections and complicated pneumonia, potentially progressing to pleural empyema in the absence of adequate management [20,21,22,23].

Despite the above, the relationship between pneumoconiosis and the risk of developing pleural empyema remains incompletely elucidated. The present study was designed to ascertain whether pneumoconiosis augments the risk of pleural empyema. Identification of such an association could serve to enhance clinical awareness regarding the risk of pleural empyema in patients with pneumoconiosis, promote the early initiation of preventive interventions, and mitigate its severe respiratory consequences.

## 2. Materials and Methods

### 2.1. Data Source

The National Health Insurance (NHI) system of Taiwan, established in 1995, provides coverage to more than 99% of the population and contracts with more than 97% of healthcare facilities. In order to facilitate scholarly inquiry, the Ministry of Health and Welfare in Taiwan established the Health and Welfare Data Science Center to oversee the NHI database. This comprehensive repository contains detailed medical records of all insured individuals dating back to 1995.

### 2.2. Ethics and Consent

The Health and Welfare Data Science Center implemented encryption protocols for personal and confidential information before data release. The research team obtained lawful access to the NHI data from 2009 to 2020. The Institutional Review Board of China Medical University Hospital (CMUH-113-REC1-145) granted approval for this study.

### 2.3. Case and Control Cohorts

All patients newly diagnosed with pneumoconiosis between 2009 and 2020 were included in this study. The definition of pneumoconiosis was based on the International Classification of Diseases (ICD) codes (ICD-9-CM 500–505 and ICD-10-CM J60–J65). Specifically, a patient was identified as having pneumoconiosis if the relevant ICD code was recorded in at least one inpatient claim or in two or more outpatient claims, a coding approach widely adopted in NHIRD-based studies. Individuals with a diagnosis of pleural empyema preceding their pneumoconiosis diagnosis were excluded from the study.

The previously identified pneumoconiosis cohort served as the basis for establishing a 1:4 matched control cohort. Matching criteria included age, sex, and index year, to ensure the cohorts were comparable. For individuals without pneumoconiosis, the index date was randomly assigned within their observable follow-up period and anchored to the matched index year to maintain temporal comparability. Individuals with a diagnosis of pleural empyema before the index date were also excluded from the control group. Subsequently, each patient in the pneumoconiosis cohort was frequency matched with four individuals without pneumoconiosis, selected for their identical age and sex, and with no prior history of pleural empyema in either group. Comparable methodologies have been described in several studies [9,24,25,26]. The validity of using ICD codes for disease classification in Taiwan’s NHI system is well-established, with positive predictive values reported between 80 and 99% in most investigations [27].

**Figure 1:**
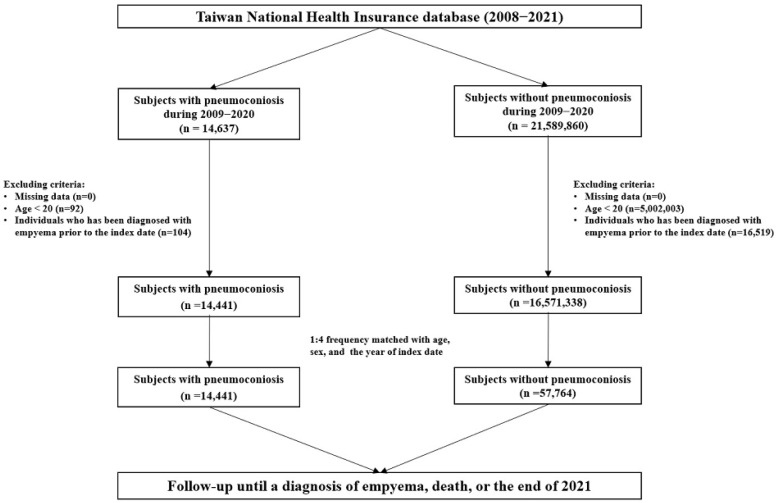
Flowchart of study subject selection.

The follow-up period for both the pneumoconiosis and control cohorts extended from the index diagnostic date until the earliest of the following events: the occurrence of pleural empyema, withdrawal from the NHI system, mortality, or the censoring date of 31 December 2021 (Figure 1).

### 2.4. Study Objectives and Covariates

Pleural empyema incidence, identified using ICD-9-CM 510 and ICD-10-CM J86, was the primary outcome. In addition, TB-related empyema was defined as tuberculosis diagnoses (ICD-9-CM 010–018 and ICD-10-CM A15–A19) occurring within three months before or after the empyema index date. Covariates considered in the study included age, sex, comorbidities, medication use, and healthcare utilization. The following relevant comorbidities were identified before the index date for statistical adjustments: hypertension (ICD-9-CM 401–405 and ICD-10-CM I10–I16), diabetes mellitus (ICD-9-CM 250 and ICD-10-CM E08–E13), hyperlipidemia (ICD-9-CM 272 and ICD-10-CM E78), heart failure (HF; ICD-9-CM 428 and ICD-10-CM I50), asthma/COPD (ICD-9-CM 491–493, 496 and ICD-10-CM J41–J45), gastroesophageal reflux disease (GERD; ICD-9-CM 530.11, 530.81 and ICD-10-CM K21), chronic liver disease (CLD; ICD-9-CM 571 and ICD-10-CM K70–K77), chronic kidney disease (CKD; ICD-9-CM 585 and ICD-10-CM N18), rheumatic disease (ICD-9-CM 279, 710.0, 714.0 and ICD-10-CM D80–D84; M05, M06, M32), pneumothorax (ICD-9-CM 512 and ICD-10-CM J93), and malignancy (ICD-9-CM 140–208 and ICD-10-CM C00–C96). Systemic corticosteroid users were defined as patients with ≥28 cumulative prescription days of oral or parenteral corticosteroids prior to the index date. The definitions for pleural empyema and comorbidities adhered to the same criteria as pneumoconiosis, requiring at least one hospitalization or two outpatient diagnoses for classification. We further incorporated healthcare utilization variables—including the number of outpatient visits and inpatient admissions during the year prior to the index date.

### 2.5. Statistical Analysis

The distribution of age, sex, comorbidities, medication use, and healthcare utilization across the study groups was compared using chi-square tests and independent samples *t*-tests. The cumulative incidence of pleural empyema in the pneumoconiosis and control cohorts was estimated via Kaplan–Meier survival analysis and statistical significance was assessed using log-rank tests. Cox proportional hazards regression models, including univariate and multivariate analyses, were employed to estimate crude and adjusted hazard ratios with corresponding 95% confidence intervals (CIs). To account for the matched study design, both stratified Cox models by matched sets and Cox models with clustered sandwich standard errors were additionally performed. The proportional hazards assumption was examined using Cox–Snell residuals to evaluate overall model fit. To address the competing risk of death, Fine–Gray subdistribution hazard models were also conducted in addition to the cause-specific Cox models. A series of sensitivity analyses were further performed, including (1) a propensity score–matched analysis (1:1) incorporating demographics, comorbidities, medication use, and healthcare utilization; (2) E-value calculations to quantify the potential influence of unmeasured confounding; and (3) a causal mediation analysis treating asthma/COPD as potential mediators. Subgroup analyses were conducted according to age, sex, comorbidity status, corticosteroid use, and healthcare utilization. Interaction terms (e.g., pneumoconiosis × age group) were evaluated to explore effect modification, and interaction *p*-values were reported. Absolute risk measures were estimated, including five-year cumulative incidence and absolute risk differences. All statistical analyses were conducted using SAS software version 9.4 (SAS Institute, Inc., Cary, NC, USA), with a significance level of *p* < 0.05.

## 3. Results

The present study included a total of 14,441 patients with pneumoconiosis and 57,764 control individuals without the disease. Table 1 presents the baseline characteristics of the study population. While age and sex distributions were similar between the two groups, patients with pneumoconiosis exhibited a significantly higher prevalence of several comorbidities: HF (10.0% vs. 7.3%, *p* < 0.001), asthma/COPD (48.5% vs. 23.1%, *p* < 0.001), GERD (18.9% vs. 14.8%, *p* < 0.001), CLD (17.1% vs. 15.4%, *p* < 0.001), rheumatic disease (2.8% vs. 1.7%, *p* < 0.001), and pneumothorax (1.09% vs. 0.24%, *p* < 0.001). Furthermore, corticosteroid use was significantly more common in the pneumoconiosis group (27.1% vs. 18.0%, *p* < 0.001). In addition, healthcare utilization was more frequent in pneumoconiosis group. The mean duration of follow-up was 5.45 ± 3.69 years for the pneumoconiosis cohort and 5.73 ± 3.60 years for the control group. The standardized mean differences for age and sex confirmed successful matching, while several comorbidities showed meaningful imbalances, justifying multivariable adjustment.

Our findings revealed a significantly elevated incidence of pleural empyema in the pneumoconiosis cohort compared to the control cohort (2.33 vs. 1.02 per 1000 person-years, respectively; Table 2). This corresponds to a 2.27 times greater risk of developing pleural empyema among patients with pneumoconiosis compared to individuals without the condition. In Cox models accounting for the matched design—using both stratified analyses by matched sets and models with clustered sandwich standard errors—results remained consistent (Supplementary data). Following adjustment for age, sex, comorbidities, and medication use, aHR for pleural empyema development in patients with pneumoconiosis was 1.79 (95% CI = 1.47–2.18), confirming a significantly increased risk. The proportional hazards assumption was satisfied based on Cox–Snell residual diagnostics (Supplementary data). In Fine–Gray competing-risk models treating death as a competing event, pneumoconiosis remained significantly associated with pleural empyema, yielding subdistribution hazard ratios comparable to the primary Cox models (Supplementary data). Furthermore, individuals with pneumoconiosis exhibited a significantly higher risk of developing TB-related empyema compared with those without pneumoconiosis, indicating that the elevated empyema risk associated with pneumoconiosis persists even when focusing specifically on TB-related cases (Supplementary data).

Consistently across subgroup analyses, patients with pneumoconiosis demonstrated a higher likelihood of developing pleural empyema, irrespective of age group, sex, presence of comorbidity, medication use, or healthcare utilization (Table 3). Remarkably, this risk was more pronounced in younger individuals (aHR = 2.57, 95% CI = 1.73–3.82). Men with pneumoconiosis exhibited a significantly elevated risk compared to men without the condition (aHR = 1.80, 95% CI = 1.47–2.19). In the subgroup of pneumoconiosis patients without comorbidities, the risk of pleural empyema was substantially higher than in the control cohort (aHR = 3.66, 95% CI = 2.52–5.31), and it remained significant among those with at least one comorbidity (aHR = 1.89, 95% CI = 1.54–2.33). Similarly, pneumoconiosis patients who did not use corticosteroids had a markedly increased risk of pleural empyema compared to the control cohort (aHR = 1.88, 95% CI = 1.49–2.36), while a significant risk persisted among those who used corticosteroids (aHR = 1.57, 95% CI = 1.08–2.29). Several interaction terms between pneumoconiosis and subgroup variables reached statistical significance, supporting effect modification in age, comorbidity status, and healthcare utilization.

Examining the combined influence of pneumoconiosis and each individual comorbidity demonstrated a cumulative effect on the incidence of pleural empyema (Table 4). Remarkably, patients with pneumoconiosis comorbid with pneumothorax, HF, CKD, diabetes, and asthma/COPD showed the highest rates of pleural empyema (7.97, 3.94, 3.54, 2.94 and 2.91 per 1000 person-years, respectively).

In additional analyses, a 1:1 propensity score–matched cohort demonstrated effect estimates consistent with the main findings, supporting the robustness of the association (Supplementary data). The E-value for the observed aHR indicated that only a strong unmeasured confounder could fully explain the association (Supplementary data). A causal mediation analysis further suggested that asthma/COPD only partially mediated the association between pneumoconiosis and empyema, indicating that other mechanisms contribute to the increased risk (Supplementary Data).

**Figure 2:**
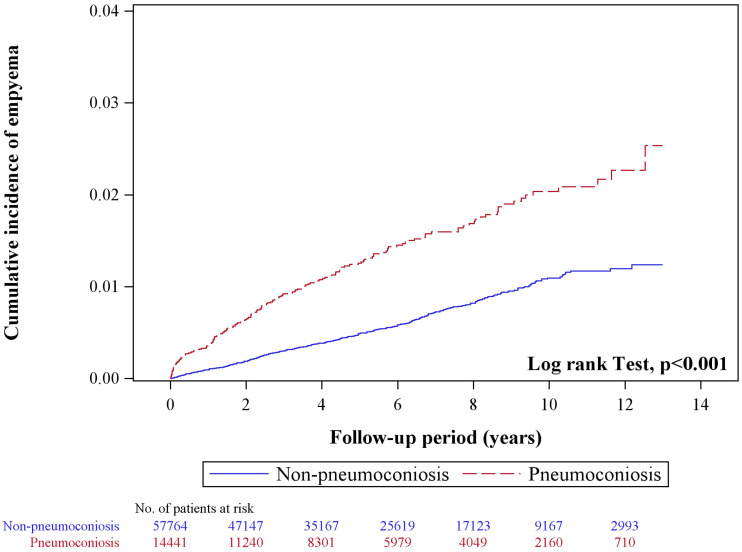
Cumulative incidence curves of pleural empyema among individuals with and without pneumoconiosis.

Figure 2 illustrates the Kaplan–Meier survival analysis, demonstrating a significantly higher cumulative incidence of pleural empyema in the pneumoconiosis group compared to the control group (*p* < 0.001). The survival curves began to diverge early during the follow-up period and continued to separate over time, emphasizing the long-lasting effect of pneumoconiosis on the development of pleural empyema. The five-year cumulative incidence was markedly higher in the pneumoconiosis cohort, further highlighting the sustained and clinically meaningful elevation in long-term risk (Supplementary Data).

## 4. Discussion

Utilizing a large retrospective cohort design and Taiwan’s National Health Insurance database, this study revealed a significantly increased risk of developing pleural empyema in patients with pneumoconiosis compared to their counterparts without the condition. Even after adjusting for age, sex, comorbidities, and corticosteroid use, aHR remained at 1.79 (95% CI = 1.47–2.18), representing near double the risk. Importantly, this heightened risk persisted across multiple subgroups, including individuals without comorbidities (aHR = 3.66) and those not using corticosteroids (aHR = 1.88), underscoring the independent contribution of pneumoconiosis to the development of pleural empyema.

Pleural empyema, a severe and potentially life-threatening complication of pneumonia, is defined by the collection of purulent fluid within the pleural cavity [28,29,30]. Rapid diagnosis and appropriate management are vital to minimize morbidity and mortality [31,32,33]. The increased incidence of pleural empyema observed in pneumoconiosis patients in this study indicates a heightened vulnerability to severe pneumonia and subsequent pleural complications. This underscores the importance of a high index of clinical suspicion and timely intervention in pneumoconiosis patients presenting with respiratory infections, especially considering the increased risk even among younger individuals and those without significant comorbidities.

Beyond inducing pulmonary fibrosis, pneumoconiosis is known to significantly alter pleural anatomy and physiology. Common structural changes in advanced disease include pleural thickening, effusion, and invagination [17]. These pleural abnormalities, frequently resulting from chronic inflammation and fibrotic remodeling triggered by sustained dust exposure, disrupt the normal balance of pleural fluid production and absorption [16]. The compromised pleural microenvironment associated with pneumoconiosis may facilitate secondary bacterial invasion and infection, particularly in the context of pneumonia [21]. Furthermore, impaired clearance of inhaled pathogens—attributable to dysfunctional alveolar macrophages and a disrupted alveolar–capillary barrier—enhances susceptibility to severe pulmonary infections [18]. When inflammatory processes in the lung extend into the pleural space, the pre-existing pleural fibrosis and fluid stasis can predispose parapneumonic effusions to progress into complex parapneumonic effusions or loculated empyema [16]. The formation of fibrin mesh and impaired pleural fluid drainage, driven by inflammatory mediators like TNF-α and fibrin-depositing pathways, are key in this process [16]. Furthermore, the systemic immunosuppression and coexisting health issues often present in pneumoconiosis patients elevate their risk for uncontrolled pleural infections [20]. Remarkably, pneumonia has been identified as the primary cause of acute respiratory failure in coal workers’ pneumoconiosis, often leading to poor outcomes in mechanically ventilated individuals [19]. These observations support the hypothesis that pneumoconiosis contributes to the development of pleural empyema via a multifactorial pathway encompassing pleural structural alterations, compromised immunity, enhanced bacterial translocation, and an imbalance in fibrinolysis. Collectively, pneumoconiosis predisposes individuals to pleural empyema not only by increasing the likelihood and severity of pneumonia but also by establishing a pleural environment conducive to bacterial persistence and impaired resolution. Consequently, heightened clinical vigilance and the implementation of early intervention strategies are warranted in this high-risk population.

Given that patients with pneumoconiosis often represent socially and medically vulnerable populations, frequently retired or unemployed workers from high-risk industries with potentially limited healthcare access and a higher burden of chronic comorbidities such as cardiovascular disease [7,8,9,10], CVD [11,12], CKD [24], and mental disorders [26], the elevated risk of pleural empyema observed in this group has significant public health implications. Targeted strategies, including regular pulmonary follow-up, vaccination initiatives, and early infection control, are crucial. Additionally, policy changes to guarantee accessible, high-quality care for individuals with pneumoconiosis are necessary to reduce health inequalities and improve their overall well-being.

The use of a large and thoroughly characterized nationwide database, coupled with extensive follow-up information, constitutes a major advantage of this study. Taiwan’s NHI system’s broad coverage helps to minimize selection bias and ensures a sample representative of the overall population [34,35]. Additionally, the careful matching of cohorts aided in controlling for potential confounding factors, bolstering the reliability of our results. However, several limitations inherent to claims-based research should be acknowledged. First, disease identification relied on ICD codes, which may cause minor misclassification despite the high validity of Taiwan’s NHI database. Second, information on lifestyle and behavioral confounders—such as smoking habits, alcohol consumption, occupational exposure after diagnosis, vaccination status, dental infection history, and prior pneumonia episodes—was unavailable in the administrative data. These unmeasured factors may influence both pneumoconiosis severity and susceptibility to pleural empyema; however, most would be expected to bias the results toward stronger positive associations, suggesting that the true effect may be equal or greater than our estimates. Third, surveillance bias cannot be entirely excluded, as patients with pneumoconiosis may have more frequent healthcare contact. Nonetheless, the incorporation of outpatient and inpatient utilization in the adjusted models mitigates this concern. Nevertheless, the consistency of our findings across multiple sensitivity and subgroup analyses suggests that the observed associations are unlikely to be fully explained by these unmeasured variables. Finally, laboratory parameters and imaging findings were not captured in the database, precluding direct assessment of disease activity or radiologic severity. In addition, due to the inherent limitations of ICD coding practices in the NHI database, pneumoconiosis subtypes (e.g., silicosis, asbestosis, coal workers’ pneumoconiosis) could not be totally distinguished. This constraint reflects the real-world nature of administrative data, and future studies are warranted to explore potential differences among pneumoconiosis subtypes.

## 5. Conclusions

Our findings indicate that patients with pneumoconiosis face a significantly greater risk of developing pleural empyema, likely driven by factors including increased susceptibility to pneumonia, impaired immune function, and structural damage to the lungs. Therefore, intensified monitoring for pleural complications in pneumoconiosis patients who develop respiratory infections may lead to earlier diagnosis and a reduction in the negative outcomes associated with pleural empyema.

## Figures and Tables

**Table 1:** Characteristics for individuals with and without pneumoconiosis.

	Pneumoconiosis					
	No		Yes			
	N = 57,764		N = 14,441			
	n	%	n	%	*p*-Value ^†^	SMD
Age					>0.999	
20–64	20,928	36.2	5232	36.2		<0.001
65–74	17,276	29.9	4319	29.9		<0.001
≥75	19,560	33.9	4890	33.9		<0.001
Mean ± SD	67.8	±12.7	67.9	±12.6	0.476	0.007
Gender					>0.999	<0.001
Women	8816	15.3	2204	15.3		
Men	48,948	84.7	12,237	84.7		
Comorbidity						
Hypertension	31,573	54.7	7401	51.3	<0.001	0.068
Diabetes mellitus	15,705	27.2	3174	22.0	<0.001	0.121
Hyperlipidemia	19,624	34.0	4445	30.8	<0.001	0.068
HF	4195	7.26	1444	10.0	<0.001	0.098
Asthma/COPD	13,314	23.1	7010	48.5	<0.001	0.552
GERD	8537	14.8	2734	18.9	<0.001	0.111
CLD	8877	15.4	2466	17.1	<0.001	0.046
CKD	4424	7.66	1016	7.04	0.011	0.024
Rheumatic disease	963	1.67	405	2.80	<0.001	0.077
Pneumothorax	141	0.24	157	1.09	<0.001	0.104
Malignancy	4909	8.50	1255	8.69	0.460	0.007
Medication						
Corticosteroid	10,409	18.0	3916	27.1	<0.001	<0.001
Healthcare utilization in the past year						
Outpatient visits					<0.001	
0–14	26,019	45.0	4549	31.5		0.281
15–28	16,444	28.5	4732	32.8		0.093
≥29	15,301	26.5	5160	35.7		0.201
Mean ± SD	21	18.4	26.3	19.8	<0.001	0.278
Inpatient admission					<0.001	0.713
0	47,327	81.9	7240	50.1		
≥1	10,437	18.1	7201	49.9		
Mean ± SD	0.33	1.00	0.84	1.33	<0.001	0.433

CLD, chronic liver disease; CKD, chronic kidney disease; COPD, chronic obstructive pulmonary disease; CVD, cerebrovascular disease; GERD, gastroesophageal reflux disease; HF, heart failure; SD, standard deviation; SMD, standardized mean difference, a negligible imbalance in potential confounders between the two groups was indicated by SMD < 0.1; ^†^ Chi-squired test and *t*-test; Pneumoconiosis patients and non-pneumoconiosis individuals were frequency matched at a 1:4 ratio based on age, sex, and index year.

**Table 2:** Risk factor analyses for pleural empyema among all study individuals.

	Event	PY	Rate ^†^	Crude HR (95% CI)	Adjusted HR ^#^ (95% CI)
Pneumoconiosis					
No	339	331,097	1.02	1 (Reference)	1 (Reference)
Yes	183	78,663	2.33	2.27 (1.89–2.71) ***	1.79 (1.47–2.18) ***
Age					
20–64	135	173,621	0.78	1 (Reference)	1 (Reference)
65–74	166	129,815	1.28	1.64 (1.31–2.06) ***	1.38 (1.09–1.75) **
≥75	221	106,324	2.08	2.64 (2.13–3.28) ***	1.96 (1.55–2.48) ***
Gender					
Women	16	65,115	0.25	1 (Reference)	1 (Reference)
Men	506	344,645	1.47	5.96 (3.63–9.81) ***	5.12 (3.11–8.44) ***
Comorbidity					
Hypertension	291	200,916	1.45	1.29 (1.09–1.54) **	0.90 (0.74–1.10)
Diabetes mellitus	149	92,534	1.61	1.35 (1.12–1.64) **	1.32 (1.07–1.63) **
Hyperlipidemia	145	128,937	1.12	0.83 (0.68–1.01)	0.72 (0.58–0.89) ***
HF	51	21,565	2.36	1.90 (1.42–2.54) ***	1.07 (0.79–1.46)
Asthma/COPD	210	96,483	2.18	2.16 (1.81–2.57) ***	1.35 (1.11–1.65) **
GERD	74	52,623	1.41	1.10 (0.86–1.41)	0.90 (0.69–1.16)
CLD	78	55,984	1.39	1.09 (0.86–1.39)	1.03 (0.80–1.32)
CKD	58	20,066	2.89	2.37 (1.80–3.11) ***	1.72 (1.28–2.29) ***
Rheumatic disease	10	6357	1.57	1.22 (0.65–2.28)	1.05 (0.56–1.98)
Pneumothorax	5	1028	4.87	3.71 (1.54–8.96) **	1.82 (0.75–4.42)
Malignancy	49	24,290	2.02	1.60 (1.20–2.16) **	1.19 (0.88–1.61)
Medication					
Corticosteroid	130	62,381	2.08	1.81 (1.49–2.22) ***	1.26 (1.01–1.57) *
Outpatient visits					
0–14	186	187,349	0.99	1 (Reference)	1 (Reference)
15–28	162	119,208	1.36	1.36 (1.10–1.68) **	1.01 (0.81–1.26)
≥29	174	103,202	1.69	1.68 (1.37–2.07) ***	0.98 (0.77–1.25)
Inpatient admission					
0	333	335,120	0.99	1 (Reference)	1 (Reference)
≥1	189	74,640	2.53	2.51 (2.10–3.00) ***	1.64 (1.34–2.00) ***

CI, confidence interval; CLD, chronic liver disease; CKD, chronic kidney disease; COPD, chronic obstructive pulmonary disease; GERD, gastroesophageal reflux disease; HF, heart failure; HR, hazard ratio; PY, person-years; ^†^ Incidence rate per 1000 person-years; ^#^ Multivariable analysis including age, gender, comorbidity, and medication; * *p* < 0.05, ** *p* < 0.01, *** *p* < 0.001.

**Table 3:** Incidences and hazard ratios of pleural empyema for individuals with and without pneumoconiosis by age, gender, comorbidity, and medication.

	Pneumoconiosis								
	No			Yes					
	Event	PY	Rate ^†^	Event	PY	Rate ^†^	Crude HR (95% CI)	Adjusted HR ^#^ (95% CI)	Interaction *p*
Age									<0.001
20–64	72	140,584	0.51	63	33,037	1.91	3.69 (2.63–5.18) ***	2.57 (1.73–3.82) ***	
65–74	109	104,331	1.04	57	25,484	2.24	2.14 (1.55–2.95) ***	1.68 (1.19–2.38) **	
≥75	158	86,182	1.83	63	20,142	3.13	1.71 (1.27–2.29) ***	1.39 (1.01–1.90) *	
Gender									0.72
Women	11	52,448	0.21	5	12,666	0.39	1.88 (0.65–5.41)	1.46 (0.46–4.64)	
Men	328	278,649	1.18	178	65,996	2.70	2.29 (1.91–2.74) ***	1.80 (1.47–2.19) ***	
Comorbidity ^‡^									<0.001
No	69	103,167	0.67	46	17,431	2.64	3.94 (2.71–5.72) ***	3.66 (2.52–5.31) ***	
Yes	270	227,930	1.18	137	61,232	2.24	1.89 (1.54–2.32) ***	1.89 (1.54–2.33) ***	
Corticosteroid									0.539
No	264	285,616	0.92	128	61,762	2.07	2.24 (1.81–2.77) ***	1.88 (1.49–2.36) ***	
Yes	75	45,481	1.65	55	16,901	3.25	1.98 (1.40–2.81) ***	1.57 (1.08–2.29) *	
Outpatient visits									<0.001
0–14	130	161,030	0.81	56	26,319	2.13	2.64 (1.93–3.61) ***	2.10 (1.47–2.99) ***	
15–28	100	92,893	1.08	62	26,315	2.36	2.19 (1.60–3.01) ***	1.68 (1.19–2.38) **	
≥29	109	77,174	1.41	65	26,028	2.50	1.77 (1.30–2.41) ***	1.49 (1.07–2.07) *	
Inpatient admission									0.029
0	246	287,905	0.85	87	47,215	1.84	2.14 (1.68–2.74) ***	2.02 (1.57–2.61) ***	
≥1	93	43,192	2.15	96	31,448	3.05	1.43 (1.07–1.90) *	1.44 (1.06–1.95) *	

CI, confidence interval; HR, hazard ratio; PY, person-years; ^†^ Incidence rate per 1000 person-years; ^#^ Multivariable analysis including age, gender, comorbidity, and medication; ^‡^ Individuals with any comorbidity of hypertension, diabetes, hyperlipidemia, HF, asthma/COPD, GERD, CLD, CKD and rheumatic disease were classified into the comorbidity group; * *p* < 0.05, ** *p* <0.01, *** *p* <0.001.

**Table 4:** Incidences and hazard ratios of pleural empyema for individuals with and without pneumoconiosis by every comorbidity.

	Pneumoconiosis								
	No			Yes					
	Event	PY	Rate ^†^	Event	PY	Rate ^†^	Crude HR (95% CI)	Adjusted HR ^#^ (95% CI)	Interaction *p*
Hypertension									0.002
No	131	167,457	0.78	100	41,386	2.42	3.07 (2.37–3.99) ***	2.36 (1.76–3.16) ***	
Yes	208	163,640	1.27	83	37,277	2.23	1.76 (1.36–2.26) ***	1.42 (1.08–1.86) *	
Diabetes									0.698
No	234	253,505	0.92	139	63,720	2.18	2.35 (1.91–2.90) ***	1.85 (1.47–2.33) ***	
Yes	105	77,592	1.35	44	14,942	2.94	2.18 (1.53–3.09) ***	1.59 (1.09–2.32) *	
Hyperlipidemia									0.632
No	244	225,107	1.08	133	55,716	2.39	2.20 (1.78–2.71) ***	1.70 (1.35–2.15) ***	
Yes	95	105,990	0.90	50	22,947	2.18	2.43 (1.72–3.42) ***	1.99 (1.37–2.88) ***	
HF									0.929
No	310	315,118	0.98	161	73,077	2.20	2.24 (1.85–2.71) ***	1.76 (1.43–2.17) ***	
Yes	29	15,979	1.81	22	5,586	3.94	2.19 (1.26–3.81) **	2.06 (1.13–3.76) *	
Asthma/COPD									0.105
No	228	268,693	0.85	84	44,584	1.88	2.22 (1.73–2.85) ***	1.96 (1.50–2.56) ***	
Yes	111	62,404	1.78	99	34,079	2.91	1.65 (1.26–2.17) ***	1.58 (1.19–2.11) **	
GERD									0.241
No	291	290,960	1.00	157	66,177	2.37	2.37 (1.95–2.88) ***	1.89 (1.53–2.34) ***	
Yes	48	40,137	1.20	26	12,486	2.08	1.73 (1.08–2.80) *	1.32 (0.79–2.21)	
CLD									0.219
No	286	287,145	1.00	158	66,630	2.37	2.38 (1.96–2.89) ***	1.84 (1.49–2.28) ***	
Yes	53	43,952	1.21	25	12,033	2.08	1.73 (1.07–2.78) *	1.44 (0.86–2.41)	
CKD									0.055
No	294	314,705	0.93	170	74,988	2.27	2.42 (2.00–2.92) ***	1.93 (1.57–2.38) ***	
Yes	45	16,392	2.75	13	3675	3.54	1.29 (0.70–2.40)	0.99 (0.52–1.89)	
Rheumatic Dz									0.832
No	334	326,509	1.02	178	76,894	2.31	2.26 (1.88–2.71) ***	1.79 (1.47–2.18) ***	
Yes	5	4588	1.09	5	1769	2.83	2.59 (0.75–8.96)	1.75 (0.44–6.88)	
Pneumothorax									0.953
No	339	330,697	1.03	178	78,035	2.28	2.22 (1.85–2.66) ***	1.77 (1.46–2.16) ***	
Yes	0	400	0.00	5	628	7.97	-	-	
Malignancy									0.082
No	303	312,042	0.97	170	73,428	2.32	2.38 (1.97–2.87) ***	1.85 (1.51–2.28) ***	
Yes	36	19,055	1.89	13	5235	2.48	1.34 (0.71–2.53)	1.17 (0.59–2.33)	

CI, confidence interval; CLD, chronic liver disease; CKD, chronic kidney disease; COPD, chronic obstructive pulmonary disease; GERD, gastroesophageal reflux disease; HF, heart failure; HR, hazard ratio; PY, person-years; ^†^ Incidence rate per 1000 person-years; ^#^ Multivariable analysis including age, gender, comorbidity, and medication; * *p* < 0.05, ** *p* < 0.01, *** *p* < 0.001.

## Data Availability

Data are available from the Health and Welfare Data Science Center, Ministry of Health and Welfare, Taiwan. Due to legal restrictions, the data are not publicly available but can be accessed upon formal application and approval according to the center’s data-use policies.

## Supplementary Materials

The following supporting information can be downloaded at https://www.mdpi.com/article/10.3390/diagnostics15233075/s1, Supplementary Table S1. Comparison of Cox proportional hazards models after matching; Supplementary Figure S1. Cox–Snell residual plot for the multivariable Cox proportional hazards model; Supplementary Table S2. Competing-risks analysis for the association between pneumoconiosis and pleural empyema; Supplementary Table S3. Causal mediation analysis of the association between pneumoconiosis and pleural empyema using COPD/asthma as potential mediators; Supplementary Table S4. Five-year cumulative incidence of pleural empyema in individuals with and without pneumoconiosis; Supplementary Table S5. Secondary analysis: Cox proportional hazards models for tuberculosis-related empyema risk; Supplementary Table S6. Baseline characteristics of individuals with and without pneumoconiosis before and after propensity score matching; Supplementary Table S7. E-value analysis for the association between pneumoconiosis and pleural empyema after frequency matching and propensity score matching.
