# Supplementary material for: Association Between Pneumoconiosis and Pleural Empyema: A Retrospective Cohort Study
Source: Diagnostics (Basel). 2025 Dec 3;15(23):3075. doi: 10.3390/diagnostics15233075 (PMC12691826; doi:10.3390/diagnostics15233075)
Supplement: Supplementary file 1 [file diagnostics-15-03075-s001.zip › diagnostics-3904923-supplementary.pdf]

Supplementary data

Supplementary Table S1. Comparison of Cox proportional hazards models after matching.

Stratified Cox models by matched sets and Cox models using clustered sandwich standard errors were applied to account for the 1:4 matched design. Both models yielded results consistent with the main Cox analysis, confirming the robustness of the association between pneumoconiosis and empyema risk.

Table S1 Cox models accounting for matched design: stratified vs. clustered sandwich approaches

(a) Stratified Cox proportional hazards model by matched sets

	Crude HR (95% CI)	*p*-value	Adjusted HR # (95% CI)	*p*-value
Pneumoconiosis				
No	1.00 (reference)	-	1.00 (reference)	-
Yes	2.28 (1.90, 2.72)***	<0.001	1.79 (1.47, 2.18)***	<0.001

(b) Cox proportional hazards model with clustered sandwich standard errors

	Crude HR (95% CI)	*p*-value	Adjusted HR # (95% CI)	*p*-value
Pneumoconiosis				
No	1.00 (reference)	-	1.00 (reference)	-
Yes	2.27 (1.89, 2.72)***	<0.001	1.68 (1.43, 1.98)***	<0.001

Supplementary Figure S1. Cox–Snell residual plot for the multivariable Cox proportional hazards model.

The cumulative hazard of the Cox–Snell residuals approximates the 45-degree reference line, with only slight deviation at the tail, indicating an overall good model fit and no major violation of the proportional hazards assumption.

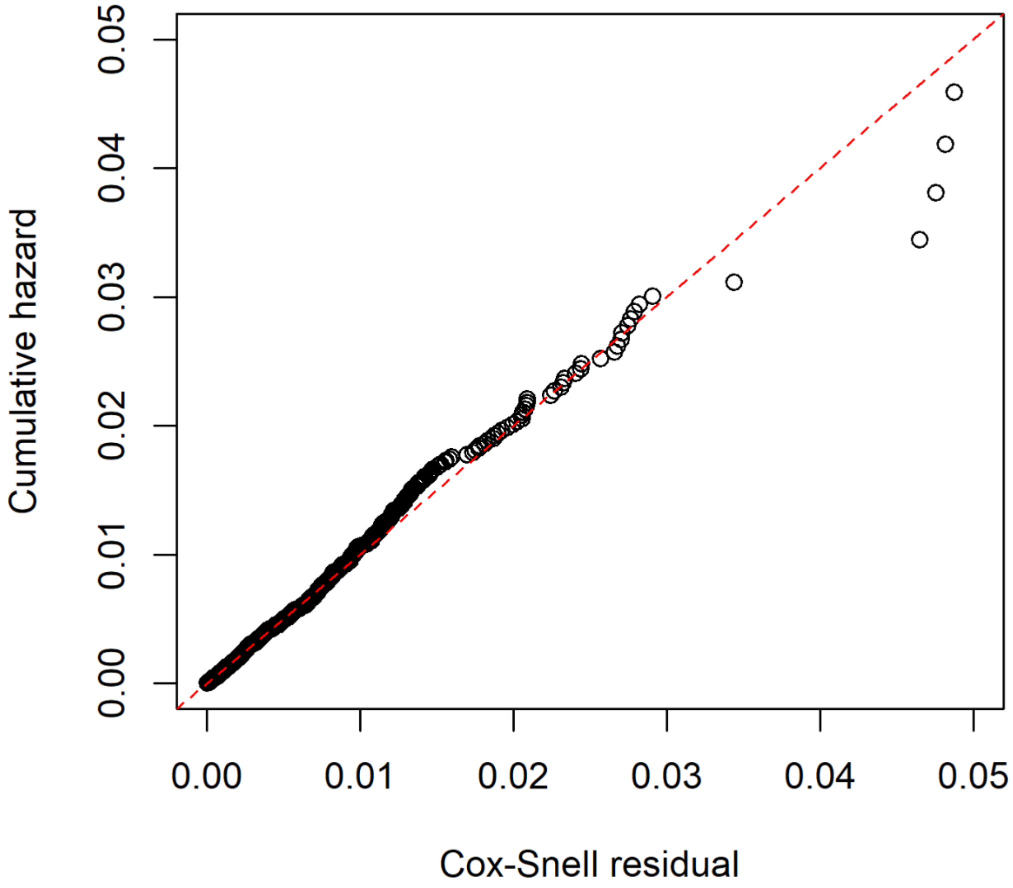

Supplementary Table S2. Competing-risks analysis for the association between pneumoconiosis and pleural empyema.

This table presents the results of the Fine–Gray subdistribution hazards models evaluating the effect of pneumoconiosis on the risk of developing pleural empyema while accounting for death as a competing event. Subdistribution hazard ratios (cSHR and aSHR) with corresponding 95% confidence intervals are reported for both unadjusted and multivariable-adjusted analyses. Adjustment variables include age, gender, comorbidities, medication use, and healthcare utilization. The Fine–Gray competing-risks approach demonstrates that pneumoconiosis remains significantly associated with an increased risk of empyema after accounting for mortality as a competing event.

Table S2 Competing risks analysis

	Pneumoconiosis	cSHR (95% CI)	p-value	aSHR[§] (95% CI)	p-value
Empyema	No	1.00 (reference)	-	1.00 (reference)	-
	Yes	2.13 (1.78, 2.55)***	<0.001	1.85 (1.52, 2.26)***	<0.001
Death	No	1.00 (reference)	-	1.00 (reference)	-
	Yes	1.29 (1.25, 1.33)***	<0.001	1.05 (1.01, 1.08)**	0.006

CI, confidence interval; SHR: subdistribution hazard ratio by using Fine-Grey model.

[#] Multivariable analysis including age, gender, comorbidity, medication, and healthcare utilization;

* p <0.05, ** p <0.01, *** p <0.001.

Supplementary Table S3. Causal mediation analysis of the association between pneumoconiosis and pleural empyema using COPD/asthma as potential mediators. This table summarizes the results of the causal mediation analysis based on the Cox proportional hazards model. The total effect, controlled direct effect (CDE), and natural direct effect (NDE) were statistically significant, whereas the natural indirect effect (NIE) was not, indicating that COPD/asthma did not mediate the relationship between pneumoconiosis and empyema.

Table S3 Causal mediation analysis of the association between pneumoconiosis and empyema, with COPD/asthma as potential mediators

Summary of Causal Mediation Effects Using Cox Proportional Hazards Model

	Estimate (95% CI)	*p*-value
Hazard Ratio Total Effect	1.95 (1.61, 2.37)***	<0.001
Hazard Ratio CDE	1.98 (1.53, 2.56)***	<0.001
Hazard Ratio NDE	1.87 (1.52, 2.32)***	<0.001
Hazard Ratio NIE	1.04 (0.97, 1.12)	0.276

CDE: Controlled direct effect, the effect of the exposure on the outcome when the mediator is held constant;

NDE: Natural direct effect, the direct effect of the exposure, not through the mediator;

NIE: Natural indirect effect, the indirect effect of the exposure that works through the mediator;

Supplementary Table S4. Five-year cumulative incidence of pleural empyema in individuals with and without pneumoconiosis.

This table presents the 5-year cumulative incidence rates of pleural empyema for pneumoconiosis and non-pneumoconiosis groups. The incidence was 0.013 in the pneumoconiosis group and 0.005 in the non-pneumoconiosis group, yielding an absolute risk difference of 0.008. These results highlight the elevated absolute risk of empyema associated with pneumoconiosis.

Table S4 5-year cumulative incidence rates

Pneumoconiosis	5-year cumulative incidence rates	Risk difference
No	0.005	0.008
Yes	0.013	

Supplementary Table S5. Secondary analysis: Cox proportional hazards models for tuberculosis-related empyema risk.

This table presents the results of the secondary analysis evaluating the association between pneumoconiosis and the risk of tuberculosis-related empyema. Incidence rates, crude hazard ratios, and adjusted hazard ratios (adjusting for age, gender, comorbidities, medications, and healthcare utilization) are shown. Individuals with pneumoconiosis exhibited a significantly higher risk of developing TB-related empyema compared with those without pneumoconiosis, indicating that the elevated empyema risk associated with pneumoconiosis persists even when focusing specifically on TB-related cases.

Table S5 Secondary analysis: Cox models for tuberculosis-related empyema risk

	Event	PY	Rate †	Crude HR (95% CI)	*p*-value	Adjusted HR # (95% CI)	*p*-value
Pneumoconiosis							
No	27	331647.55	0.08	1.00 (reference)	-	1.00 (reference)	-
Yes	24	79017.81	0.30	3.72 (2.15, 6.44)***	<0.001	2.95 (1.61, 5.39)***	<0.001

CI, confidence interval; HR, hazard ratio; PY, person-years;

† Incidence rate per 1,000 person-years;

# Multivariable analysis including age, gender, comorbidity, medication, and healthcare utilization;

* p <0.05, ** p <0.01, *** p <0.001.

Supplementary Table S6. Baseline characteristics of individuals with and without pneumoconiosis before and after propensity score matching.

This table presents demographic characteristics, comorbidities, medication use, and healthcare utilization for pneumoconiosis and non-pneumoconiosis patients prior to matching and after 1:1 propensity score matching. Standardized mean differences (SMDs) are provided to assess covariate balance, with SMD <0.1 indicating negligible imbalance between matched groups.

Table S6 Characteristics for individuals with and without pneumoconiosis before and after propensity score matching

	Before PS matching					After PS matching				
	Pneumoconiosis					Pneumoconiosis				
	No N = 16571338		Yes N = 14441			No N = 14441		Yes N = 14441		
	n	%	n	%	SMD	n	%	n	%	SMD
Age										
20–64	13652876	82.39	5232	36.23	1.064	4393	30.42	5232	36.23	0.123
65 74	1548309	9.34	4319	29.91	0.536	4498	31.15	4319	29.91	0.027
≥ 75	1370153	8.27	4890	33.86	0.661	5550	38.43	4890	33.86	0.095
Mean ±SD	47.59	17.28	67.91	12.56	1.345	69.57	12.46	67.91	12.56	0.133
Gender					0.809					0.166
Women	8205116	49.51	12237	84.74		11309	78.31	12237	84.74	
Men	8366222	50.49	2204	15.26		3132	21.69	2204	15.26	
Comorbidity										
Hypertension	4130228	24.92	7401	51.25	0.563	8437	58.42	7401	51.25	0.145
Diabetes mellitus	2111541	12.74	3174	21.98	0.246	4008	27.75	3174	21.98	0.134
Hyperlipidemia	3397015	20.50	4445	30.78	0.237	5295	36.67	4445	30.78	0.125
HF	409897	2.47	1444	10.00	0.315	1882	13.03	1444	10.00	0.095
Asthma/COPD	1933304	11.67	7010	48.54	0.878	7840	54.29	7010	48.54	0.115
GERD	2033518	12.27	2734	18.93	0.184	3568	24.71	2734	18.93	0.140
CLD	1847942	11.15	2466	17.08	0.171	3187	22.07	2466	17.08	0.126
CKD	441127	2.66	1016	7.04	0.205	1568	10.86	1016	7.04	0.134
Rheumatic disease	260547	1.57	405	2.80	0.084	626	4.33	405	2.80	0.083
Pneumothorax	27123	0.16	157	1.09	0.117	108	0.75	157	1.09	0.036
Malignancy	598360	3.61	1255	8.69	0.213	1807	12.51	1255	8.69	0.124
Medication										
Corticosteroid	1854873	11.19	3916	27.12	0.413	4880	33.79	3916	27.12	0.145
Healthcare utilization in the past year										
Outpatient visits										
0-14	10432874	62.96	4549	31.50	0.664	3453	23.91	4549	31.50	0.170
15-28	3811526	23.00	4732	32.77	0.219	4406	30.51	4732	32.77	0.049
29	2326938	14.04	5160	35.73	0.518	6582	45.58	5160	35.73	0.201
Mean ±SD	14.52	14.82	26.32	19.80	0.675	30.78	21.68	26.32	19.80	0.215
Inpatient visits					0.993					0.215
0	15034516	90.73	7240	50.14		8773	60.75	7240	50.14	
1	1536822	9.27	7201	49.86		5668	39.25	7201	49.86	
Mean ±SD	0.15	0.69	0.84	1.33	0.653	0.85	1.79	0.84	1.33	0.002
Mean follow-up time ±SD	6.47	3.53	5.45	3.69	0.283	5.07	3.55	5.45	3.69	0.103

† Chi-squired test and t-test.

Pneumoconiosis and non-pneumoconiosis patients were propensity score matched at a 1:4 ratio based on age, sex, index year, comorbidity, medication, and healthcare utilization. Patients with pneumoconiosis were matched 1:1 with non-pneumoconiosis individuals using propensity score matching through the nearest neighbor method. Initially, matching was performed to the eighth digit, and if no match was found, it was progressively relaxed to the first digit. The matching process began with a caliper width of 0.0000001, which was gradually increased to 0.1 for unmatched cases. To refine the matching, we reassessed the criteria and conducted a rematch using a greedy algorithm, ensuring that each pneumoconiosis patient was paired with the closest propensity score match.

Supplementary Table S7. E-value analysis for the association between pneumoconiosis and pleural empyema after frequency matching and propensity score matching.

This table displays incidence rates, hazard ratios, and E-values assessing the potential influence of unmeasured confounding on the observed association. The E-values for the adjusted hazard ratios indicate the minimum strength of association an unmeasured confounder would need to fully explain the pneumoconiosis–empyema relationship, supporting the robustness of the findings.

Table S7 E-value of empyema among all study individuals after frequency and propensity score matching

	Pneumoconiosis										
	No			Yes							
	Event	PY	Rate †	Event	PY	Rate †	Crude HR (95% CI)	p-value	Adjusted HR # (95% CI)	p-value	E-value § (95% CI)
Pneumoconiosis and non-pneumoconiosis patients were frequency matched at a 1:4 ratio based on age, gender, and index year.	339	331097.01	1.02	183	78662.6	2.33	2.27 (1.89, 2.71)***	<0.001	1.79 (1.47, 2.18)***	<0.001	2.98 (2.3, 3.78)
Pneumoconiosis and non-pneumoconiosis patients were propensity score matched at a 1:1 ratio based on age, gender, index year, comorbidity, medication, and healthcare utilization	126	73264.91	1.72	183	78662.60	2.33	1.38 (1.10, 1.73)**	0.006	1.29 (1.03, 1.63)*	0.029	1.91 (1.19, 2.64)

† Incidence rate per 1,000 person-years;
# Multivariable analysis including age, gender, comorbidity, medication, and healthcare utilization;
* p <0.05, ** p <0.01, *** p <0.001.
§E-values were calculated based on the adjusted hazard ratio.
